# Porfiria intermitente aguda: reporte de caso

**DOI:** 10.7705/biomedica.4767

**Published:** 2020-03-30

**Authors:** José Bustos, Ledmar Vargas, Ricardo Quintero

**Affiliations:** 1 Servicio de Neurología, Hospital San Rafael, Tunja, Colombia Hospital San Rafael Tunja Colombia; 2 Escuela de Medicina, Universidad Pedagógica y Tecnológica de Colombia, Tunja, Colombia Universidad Pedagógica y Tecnológica de Colombia Universidad Pedagógica y Tecnológica de Colombia Tunja Colombia; 3 Escuela de Medicina, Universidad de Boyacá, Tunja, Colombia Universidad de Boyacá Universidad de Boyacá Tunja Colombia; 4 Servicio de Medicina Interna, Hospital San Rafael, Tunja, Colombia Hospital San Rafael Tunja Colombia

**Keywords:** porfiria intermitente aguda, dolor abdominal, hiponatremia, polineuropatías, convulsiones, Porphyria, acute intermittent, abdominal pain, hyponatremia, polyneuropathies, seizures

## Abstract

El término 'porfiria' proviene del griego 'porphyra' y alude a un grupo heterogéneo de trastornos metabólicos causados por una deficiencia enzimática en la biosíntesis del grupo hemo. La causa de la porfiria intermitente aguda es la deficiencia de la enzima deaminasa del porfobilinógeno.

Se presenta el caso de una mujer de 40 años que presentó dolor abdominal de 10 días de evolución, trastorno hidroelectrolítico grave debido a hiponatremia e hipopotasemia, taquicardia e hipertensión arterial sistémica persistentes, por lo cual fue sometida a una laparotomía en la que no se encontró ninguna afección de origen quirúrgico, A los siete días del examen inicial, la paciente desarrolló cuadriparesia flácida aguda y presentó una crisis convulsiva tónico-clónica generalizada. Los estudios neurofisiológicos evidenciaron una polineuropatía axonal mixta, y los valores de porfobilinógeno y porfirinas en orina eran elevados. Tras diagnosticarse porfiria intermitente aguda, esta se trató con hemina, lo que estabilizó los signos clínicos y normalizó el porfobilinógeno. La prevalencia de esta enfermedad es de 1 en 2.000 personas. Tiene un patrón de herencia autosómico dominante y se manifiesta principalmente en mujeres con edades entre los 20 y los 40 años. La enfermedad cursa con síntomas neurológicos y viscerales, y se trata con la administración de hemina y dextrosa, evitando las soluciones hipotónicas por el riesgo de exacerbar la hiponatremia.

## Abstract (en)

The term 'porphyria' comes from the Greek 'porphyra'. It refers to a heterogeneous group of metabolic disorders caused by the enzymatic deficiency in the biosynthesis of the heme group. Acute intermittent porphyria is caused by a deficiency of the porphobilinogen deaminase enzyme.

A 40-year-old woman presented with abdominal pain for ten days (which required laparotomy that evidenced no surgical pathology), severe hydroelectrolytic disorder due to hyponatremia and resistant hypokalemia, persistent tachycardia and hypertension. Seven days later, she developed acute flabby quadriparesis and presented a single generalized tonic-clonic convulsive crisis. Neurophysiological studies supported mixed axonal polyneuropathy and urine results of porphobilinogen and porphyrins were elevated. After acute intermittent porphyria was diagnosed, hemin was administered, which stabilized the patient's clinical signs and normalized the porphobilinogen.

The prevalence of this entity is 1 in 2,000 people. It is an autosomal dominant disease, which affects mainly women between 20 and 40 years of age. This entity manifests with neurological and visceral symptoms. Management consists of hematin and dextrose administration avoiding hypotonic solutions because of the risk of exacerbating hyponatremia.

El término 'porfiria' proviene del griego 'porphyra', que significa púrpura, por el color rojizo que toma la orina al ser expuesta a la luz solar como consecuencia de la polimerización del porfobilinógeno ^1^. Las porfirias son un grupo heterogéneo de trastornos metabólicos causados por una deficiencia enzimática en la biosíntesis del grupo hemo, el cual se incorpora a la hemoglobina y la mioglobina, y tiene un papel importante en el metabolismo aerobio y la producción del trifosfato de adenosina *(adenosine triphosphate,* ATP) ^2^.

La porfiria intermitente aguda es causada por la deficiencia de la deaminasa de porfobilinógeno, también llamada sintasa de hidroximetilbilano, la tercera enzima en la vía de la síntesis del grupo hemo ^3^, y es la más común de las porfirias; su deficiencia tiene una prevalencia de 60 casos por cada 100.000 personas en los países del norte de Europa ^4^.

## Caso clínico

Se trata de una mujer de 24 años de edad, procedente del sur del departamento de Santander, remitida por un cuadro clínico de 10 días de evolución consistente en dolor abdominal intenso, emesis, hiporexia, estreñimiento, astenia y adinamia. Dos días antes de su ingreso, le habían practicado una laparotomía exploratoria en una institución de mediana complejidad debido a la agudización del dolor abdominal, durante la cual no se evidenciaron alteraciones en la cavidad abdominal.

La paciente refirió antecedentes de condilomatosis, anemia en estudio y alergia al ibuprofeno. Manifestó, asimismo, que la abuela materna tenía una enfermedad huérfana no especificada.

En el examen físico general, se registró una frecuencia cardíaca de 113 latidos por minuto, tensión arterial sistémica de 177/90 mm Hg y temperatura de 36,2 °C, y había presentado taquicardia durante todo el curso de la enfermedad. Las mucosas se encontraron secas, se observó una herida quirúrgica en el abdomen en adecuado estado de cicatrización, sin dehiscencias de suturas ni signos de infección local; sin embargo, tenía dolor agudo a la palpación superficial hipogastrio con contracción muscular voluntaria y sin signos de irritación peritoneal; en el examen neurológico se la encontró somnolienta.

Los resultados del examen de sangre realizado en el momento del ingreso se presentan en el cuadro 1a. En la tomografía computarizada (TC) de abdomen se observó líquido libre en la pelvis, neumoperitoneo residual, distensión del marco cólico y cambios posquirúrgicos recientes en la pared abdominal anterior.

**Cuadro 1:** Pruebas de laboratorio realizadas durante la hospitalización

a. Exámenes de ingreso		
Pruebas diagnósticas	Resultado	Referencia
Creatinina (mg/dl)	0,62	0,6-1,1
Nitrógeno ureico en sangre (BUN) (mg/dl)	10	8-20
Transaminasa oxalacética (TGO) (UI/L)	32	10-45
Transaminasa pirúvica (TGP) (UI/L)	25	10-45
Fosfatasa alcalina (UI/L)	113	44-147
Sodio (mEq/L)	118	135-145
Osmolaridad plasmática (mOsm/kg)	244	280-295
Potasio (mEq/L)	2,6	3,5-5,3
Cloro (mmol/L)	82	98-109
Magnesio (mg/dl)	0,8	1,7-2,2
Leucocitos (× 10^3^/L)	19,4	4,5-11,0
Neutrófilos (%)	83	Hasta 85
Linfocitos (%)	6	Hasta 10
Hemoglobina (g/dl)	15,6	13-16
Plaquetas (× 10^3^/L)	356	150-450
**b. Exámenes complementarios**		
**Pruebas diagnósticas**	**Resultado**	**Referencia**
Aldosterona (ng/dl)	4,53	2,52-39,2
Renina (ng/L)	1,6	1,6-27
Proteínas totales (g/dl)	9,8	6,0-8,3
Albumina (g/dl)	3,83	3,4-5,4
Hormona estimulante de la tiroides (mUI/L)	1,58	0,5-4,0
Sodio en orina (mEq/L)	178	Hasta 20
Potasio en orina (mEq/L)	90	25-125
Cloro en orina (mEq/L)	182	110-250
Creatinina en orina (mg/kg/día)	21,5	11-0
Osmolaridad de la orina (mOsm/kg)	364	<100
Glucosa plasmática (mg/dl)	81	70-140
Osmolaridad plasmática (mOsm/kg)	244	275-295
**c. Exámenes paraclínicos de porfirias**		
**Pruebas diagnósticas**	**Resultado**	**Referencia**
Uroporfirinas (µg en 24 horas)	687	Hasta 22
Coproporfirinas (µg en 24 horas)	1.239	Hasta 60
Pentacarboxilporfirinas (µg en 24 horas)	220	Hasta 3
Hexacarboxilporfirinas (µg en 24 horas)	67	Hasta 4
Heptacarboxilporfirinas (µg en 24 horas)	117	Hasta 9
Porfobilinógeno (mg en 24 horas)	5	0-2
Ácido aminolevulínico (mg en 24 horas)	9,8	1,5-7,5
**d. Exámenes de control - Día 14**		
**Pruebas diagnósticas**	**Resultado**	**Referencia**
Porfobilinógeno (mg en 24 horas)	2	0-2

Se estableció que la paciente cursaba con trastornos hidroelectrolíticos graves debido a hiponatremia hipoosmolar hipovolémica, hipopotasemia, hipomagnesemia e hipocloremia, probablemente secundarias a la hiporexia y la emesis. Sin embargo, a pesar de la reposición con fluidos durante dos días, los niveles séricos de sodio no mejoraron, lo que se interpretó como hiponatremia hipoosmolar euvolémica resistente, motivo por el que se amplió el estudio etiológico (cuadro 1b).

Al quinto día del ingreso, la paciente se quejó de parestesias y debilidad en los miembros inferiores, lo que se consideró inicialmente como una parálisis periódica hipopotasémica. Sin embargo, 48 horas después desarrolló cuadriparesia flácida arrefléxica y presentó una única convulsión tónico-clónica generalizada, la cual remitió por sí sola y se relacionó con la aparición de hipertensión arterial sistémica. Se ordenaron, entonces, una TC simple de cráneo y un electroencefalograma, con resultados normales; también, se practicaron una electromiografía y un estudio de conducción nerviosa, los cuales demostraron una polineuropatía axonal sensitivo-motora.

Al día siguiente, la paciente presentó insuficiencia respiratoria hipoxémica que requirió asistencia respiratoria mecánica invasiva. Ante su evolución tórpida, se convocó una junta médica (medicina interna, cuidado crítico y neurología) en la que se consideró el diagnóstico de porfiria intermitente aguda, dada la presencia de dolor abdominal agudo de origen no quirúrgico, taquicardia e hiponatremia persistente por síndrome de secreción inadecuada de la hormona antidiurética, episodio de convulsión que remitió por sí solo y polineuropatía (cuadro 1c).

Al noveno día del ingreso, se confirmó dicho diagnóstico y se inició el manejo con infusión de dextrosa al 10 % y 3 mg/kg diarios de hemina durante cuatro días, con lo cual se estabilizó el cuadro clínico. El control por exámenes de laboratorio 48 horas después de finalizado el tratamiento, evidenció normalización de los electrolitos y el porfobilinógeno, lo que permitió dar de alta a la paciente (cuadro 1d).

## Consideraciones éticas

La paciente autorizó la comunicación de su caso mediante un consentimiento informado que reposa en su historia clínica.

## Discusión

La prevalencia de portadores de la mutación de la porfiria intermitente aguda en la población occidental es de 1 en 2.000 personas y las agudizaciones se presentan en menos del 10 % de ellas ^5^. Su patrón de herencia es autosómico dominante, con una amplia heterogeneidad alélica ^6^.

La condición se manifiesta con síntomas neurológicos y viscerales, lo que la diferencia de otras porfirias que, además, incluyen manifestaciones dermatológicas ^7^^,^^8^. Se sabe que entre el 80 y el 90 % de los ataques agudos ocurren en mujeres en edad fértil ^5^. Las características clínicas de esta porfiria se describen en el cuadro 2. El diagnóstico debería considerarse en pacientes jóvenes que cursen con la tríada clínica de dolor abdominal, hiponatremia y convulsiones ^9^.

**Cuadro 2:** Características clínicas de la porfiria intermitente aguda ^4^^,^^6^

Manifestaciones clínicas	Prevalencia (%)
Dolor abdominal	85 a 95
Taquicardia	65 a 85
Vómitos	44 a 88
Estreñimiento	48 a 84
Dolor en otras partes	50 a 70
Parestesias	40 a 65
Síntomas psiquiátricos	40 a 55
Hipertensión arterial	35 a 55
Confusión	30 a 44
Convulsiones	10 a 30
Parálisis respiratoria	5 a 20
Diarrea	10
**Resultados anómalos en los exámenes de laboratorio**	**Prevalencia (%)**
Anemia	27
Falla renal	20 a 45
Hiponatremia grave (<125)	40
Hiponatremia	80
Elevación de transaminasas	61

Entre las posibles hipótesis sobre la patogenia de los signos neurológicos de la porfiria intermitente aguda, se sabe que la sobreproducción de algunos precursores de la porfirina, como el ácido aminolevulínico, está implicada en la disrupción de la barrera hematoencefálica. Esta enzima también actúa como una neurotoxina directa y podría contribuir a la formación de radicales libres y a inhibir la bomba de sodio y potasio (Na-K-ATPasa), promoviendo así la liberación incontrolada de glutamato. No obstante, las convulsiones también podrían explicarse por las alteraciones hidroelectrolíticas (hiponatremia o hipomagnesemia) asociadas ^10^^,^^11^.

Además, la acumulación de porfirinas en las neuronas de los núcleos supraóptico y paraventricular del hipotálamo, puede producir un síndrome de secreción inadecuada de la hormona antidiurética, con la consecuente retención de agua y dilución del sodio ^7^. Esta condición se caracteriza por presentar hiponatremia con osmolaridad plasmática baja (<270 mOsm/kg), euvolemia, osmolaridad elevada de la orina (>100 mOsm/kg), y concentración elevada del sodio en la orina (>40 mEq/L). Para su diagnóstico se deben excluir enfermedades que se le asemejan, como el hipotiroidismo y el hipocorticalismo (tríada clínica de hiponatremia, hipotensión e hipoglucemia) ^12^^,^^13^. La hiponatremia es la manifestación paraclínica más común en la porfiria intermitente aguda ^4^^,^^12^.

En cuanto a la neuropatía asociada con esta porfiria, su característica más común es la pérdida neuronal con degeneración walleriana, cuyo mecanismo aún se desconoce. Se maneja la hipótesis de que la alteración en la biosíntesis del grupo hemo conlleva la falla en el metabolismo aerobio, que afecta el transporte axonal, el cual depende en gran medida de la energía; esto, conjuntamente con la baja producción de trifosfato de adenosina, conduce a la muerte neuronal ^14^.

Cabe mencionar, como anécdota, que en el caso de esta paciente durante los días previos al tratamiento sus muestras de orina se expusieron al sol en reiteradas ocasiones y tomaban un color rojizo, lo cual concuerda con la descripción habitual de tal situación; sin embargo, la porfiria intermitente aguda puede presentarse sin que suceda este fenómeno ^15^.

El tratamiento debe comenzar por evitar los factores desencadenantes, incluidos los fármacos que puedan generarla ^5^, y la administración parenteral de 3 a 4 mg/kg de hemina durante cuatro días (la normalización del porfobilinógeno indica mejoría) ^8^, así como de infusión de solución salina al 0,9 % y dextrosa al 10 %, con el fin de controlar la actividad de la sintasa del ácido aminolevulínico, evitando las soluciones hipotónicas y la restricción hídrica (<2.000 ml/día) dado el alto riesgo de exacerbar la hiponatremia ^7^. Además, se deben tratar otros síntomas como el dolor y la hipertensión arterial ^6^^,^^15^. Se recomienda canalizar una vena grande -o pasar un catéter venoso central- para disminuir el riesgo de flebitis por el uso de hemina en los vasos pequeños ^8^^,^^16^.

Las crisis agudas pueden poner en riesgo la vida del paciente. A pesar de tener un buen pronóstico en los casos que reciben tratamiento oportuno, la mortalidad alcanza hasta el 10 %, lo que la convierte en un reto médico dado que su variabilidad clínica lleva a confundirla con muchas otras condiciones ^17^.

Al tratarse de una enfermedad huérfana, es conveniente el estudio genético del gen de la deaminasa del porfobilinógeno para caracterizar casos asintomáticos en la familia ^18^.
